# Living and Dying With COVID-19 in South Asian Low- and Middle-Income Countries

**DOI:** 10.3389/fpubh.2021.600878

**Published:** 2021-07-01

**Authors:** Narayan Gyawali, Hasan Mohammad Al-Amin

**Affiliations:** QIMR Berghofer Medical Research Institute, Brisbane, QLD, Australia

**Keywords:** LMICs, crisis, poverty, pandemic (COVID-19), South Asia

## Introduction

The COVID-19 pandemic is a continuing disaster for global health (176 million infections and 3.8 million deaths till the date 15th June, 2021) and the world economy (losses of USD 9 trillion) but it is the poorest nations that suffer the greatest burden. In the early weeks of the pandemic, many low- and middle-income countries (LMICs) used emergency measures to adopt at least some of the non-pharmaceutical mitigation measures recommended by the WHO (1) including social distancing, local and national “lockdowns” and the closure of international borders. The consequence of these restrictions was a precipitous and insupportable loss of income for many families. Although, high-income countries (HICs) have been able to support their citizens economically during these measures, LMICs have found the trade-off between public health and the broader needs of society hard to manage. In South-Asian LMICs like Nepal, Bangladesh, and India, more than 80% of the work force operates within a poorly paid informal employment sector that spans construction, agriculture, manufacturing, trade, transport, and domestic service (2). These workers and their families rely on daily earnings and unencumbered movement to maintain a hand-to-mouth existence. They have exhausted their medical and economic resources and are now undergoing major crises (3). The plethoric consequences of the pandemic and economic crash include hunger, malnutrition (4), mental illness, and suicide (5).

The second wave of transmission is now overwhelming South-Asian LMICs (6, 7) and the sheer number of infections in India (currently >400,000/day) pose a tremendous risk to neighbors such as Nepal and Bangladesh which share long, porous borders. This article advocates for recognition of the almost insurmountable obstacles that face the implementation of non-pharmaceutical interventions for COVID-19 management in Asian LMICs and calls for a global commitment to the equitable distribution of therapeutants and vaccines.

## Challenges to the COVID-19 Response in South-Asian LMICs

The recorded incidence of COVID-19 cases and deaths in many high-income countries (HICs) such as the USA and Europe recently exceeded that of Asian LMICs (7). The initial severity of the pandemic in HICs partly resulted from SARS-COV-2's rapid establishment in those countries with open borders and busy global and national transport networks. COVID related admissions to the UK's hospital reached a critical stage in many regions with a new coronavirus patient being admitted every 30 s (8). However, public health systems on the verge of collapse initially survived following robust contact tracing, wider testing and national “shelter at home” campaigns supported by overwhelming government aid. By the beginning of 2021, the immediate threat to many HICs had been mitigated by improvements in clinical management and drug treatments, and rapidly expanding vaccine programs. In contrast, some of the initial impact on LMICs may have been masked by a lack of testing capacity and the fact that most deaths in LMICs occur at home without notification of an attributable cause. In many Asian LMICs, human densities, crowded housing, the precarious nature of people's livelihoods, and the lack of government resources have challenged community compliance with “shelter at home” campaigns. With iniquitous access to public health services, ventilators, oxygen, therapeutants, and vaccines, the number of COVID-19 cases in LMICs is now greatly exceeding those recorded in HICs (7, 9). In many Asian LMICs, testing capacity remains inadequate, but in India there appear to be 4 times more daily cases now, than in their first transmission peak (September 2020). The current infection rates in neighboring Nepal and Bangladesh have also doubled their past records (7). Governments themselves have also exacerbated and facilitated transmission in some circumstances. In beginning of 2021, political leaders in Nepal organized mass rallies immediately prior to the second surge of the pandemic and, in India, the populist government endorsed attendance at religious festivals and political rallies (10). It is not known whether constantly evolving new variants are contributing to these totals but, in recent months, variants isolated in the United Kingdom (B.1.1.7), South Africa (B.1.351), and India (B.1.617) have all been implicated as causing local and regional transmission spikes (11, 12).

The WHO details nine “pillars” to support the global response to COVID-19 (1). These are intended to limit transmission, care for the affected and protect essential health services. The WHO acknowledges that these measures will cause significant stresses on society, the economy and public health and it urges mitigation of those effects through the protection of food access, livelihoods, and essential services (1). In this respect, LMICs are challenged on every front: They do not have the infrastructure necessary to provide intensive care for large numbers of patients with respiratory failure (9) and they do not have access to the diagnostics and management systems that are required for tracking COVID-19 with any precision or for overseeing the implementation of spatially targeted non-pharmaceutical interventions. LMICs already carry the greatest burden of other communicable infectious diseases such as tuberculosis, malaria, HIV/AIDS, dengue, and diarrhea (13). The immense stresses that these existing diseases place on already limited public health resources have now been eclipsed by the demands of the COVID-19 pandemic. Health outcomes and the management of all other infectious and chronic diseases are worsening.

In many Asian LMICs, the number of ICU beds is far less than required, access to drug treatments such as dexamethasone is limited, the supply of therapeutic oxygen and ventilators is inadequate (9). There are predictions of >1.5 M COVID-19 related deaths in India by August 2021 (14). The country's capacity to export vaccines abroad is in doubt, leaving neighboring countries fully exposed. In Nepal, the Ministry of Health warns that COVID-19 assigned beds are fully occupied, and the infection rate is beyond the capacity of the health system as patients queue for beds and oxygen (15) Bangladesh has a similar problem – for a population of almost 170 M people there are just 1,200 ICU beds - mostly in the private health sector (9).

## The Realities of COVID-19 Management Resources in LMICs

As an example of the resources available to support beleaguered communities in HICs, Australia spends US$5000 on health care per capita annually and in 2020-21, expenditure on COVID-19 is expected to peak at US$ 65 billion (16) LMICs have no such resources: Bangladesh spends US$32 per capita on health care annually, that is 0.6% of the money committed for Australians, and implemented an emergency COVID-19 response programme worth 1% of GDP or US$ 3.4 billion (2), in 2020. Those sorts of funds cannot support LMICs communities through extended periods of lockdown, social distancing, and unemployment.

Non-pharmaceutical interventions have proven highly effective in COVID-19 pandemic control, in HICs. Slowdown of virus transmission due to lockdown helped them to maintain surge capacity within the healthcare system, and provided them with the opportunity to build up health care capacity and resources required for the provision of critical care, the implementation of mass testing and contact tracing, or the development and procurement of diagnostics, therapeutics and vaccines.

In contrast, the larger proportion of people from LMICs have completely different demographics and livelihoods. Many cities, such as Delhi, Dhaka, and Kathmandu are characterized by their crowded transport, living and working conditions - most earnings are unreliable and insecure and consist of carrying out manual work in exchange for low wages, yet wage earners must retain their mobility and endure close physical proximity to others, working in confined spaces regardless of the associated risks. More than 70% of urban households in Bangladesh lost the majority of their income within days of lockdown being imposed (17).

LMICs have no choice of shelter at home, working from home, online education, and online shopping. At the beginning of the pandemic, cognizant of the overwhelming challenges that lay ahead, many LMICs did act quickly to close borders and limit virus immigration. Although, lockdowns and boarder closure have slowed down the transmission of the virus at the beginning, faced with increasing transmission, little capacity for testing and contact tracing, their benefits in the long run are less clear. The simplest of mitigating measures have proved hard to implement. Even regular and effective handwashing is difficult among LMIC households that share outdoor water supplies and latrines. For many LMICs, the only pragmatic approach to the pandemic may be to focus on implementing the basic interventions of hand washing, disinfection of surfaces, the use of masks, and staying at home when symptomatic. Even those measures will require tremendous investment in community engagement, accurate public health messaging and the provision of resources such as safe water and handwashing supplies (17).

## Vaccines – Accessibility and Iniquity

Twelve months after the pandemic's start there have been enormous global efforts to combat COVID-19. Multiple new vaccines, of varying efficacy, have been developed and approved for use in record time and 1.3 billion people have received at least one dose (18). However, in many LMICs without vaccine manufacturing capacity, inoculation programs have stalled and vaccine procurement through philanthropic organizations such as the Global Alliance for Vaccines (GAVI) is challenged by the individual self-interest of some HICs (19). Whilst the larger human population remains unvaccinated, COVID-19 continues to rage and the resulting international travel restrictions continue to devastate economies. Moreover, the continuing process of mutation and selection poses a risk even to those HICs with high vaccine coverage. Variants such as B.1.351 (isolated in South Africa), P.1 and P.2 (isolated in Brazil) share “escape mutations” that reduce their neutralization by the human antibodies generated by different variants or some vaccines (20). The longer that LMICs remain unvaccinated, the greater the risk to vaccine efficacy globally.

## Call for Global Support

Unable to afford sustained lockdowns, many communities within LMICs have had to make a choice between earning a wage and contributing reluctantly to COVID-19 transmission. Although, borders can be opened and closed with ease, most LMIC governments find national distancing measures impossible to justify or maintain because of the impact they have on the urban poor. Ultimately the world must reopen, because trade, tourism and the remittance incomes that come from emigrant employment are essential to many countries' social and economic well-being. As the pandemic and the economic crisis continue, the amount of money migrant workers send home is projected to decline by 14% by 2021, compared to the pre-COVID levels in 2019 (21).

Although, we have come to expect that the management of epidemic diseases with disproportionate burdens in LMICs is funded by coalitions of governments, banks, philanthropists, and charities, the additional funding now required for the COVID-19 response is staggering. The World Bank, in addition to its US$12 billion funding to developing countries for them to purchase and distribute COVID-19 vaccines, tests and treatment, is making available a further $160 billion to assist LMICs in mitigating the health, economic, and social shocks countries are facing (2). Organizations such as GAVI have seen billions of dollars in extra donations from its members but much more will be required if these initiatives are to have an enduring impact, not only on the health sector but also for mitigating the expected economic downturn.

In the longer term, LMICs need to develop their own capacity to manufacture and distribute vaccines. However, more than half of the population in LMICs is rural area, earning < USD 3/day, are dependent on primary health centers (2). So, production of low-cost vaccines and strengthening primary health centers is critical for them, but LMICs are challenged by their limited capacity for manufacturing test kits, limited budgets for equipment and reagents, and scant ability to make competitive bids for global supplies. Intellectual property (IP), complete or partial waiver, could be an effective way to massively scale up manufacturing of diagnostic and therapeutic needs (22). More than 100 developing nations have proposed IP amendments. HICs such as Australia, the UK, the EU did not approve those proposals.

## Conclusions

Connectivity and cross-border movements have been fundamental to economic growth across the world, but they have also facilitated the pandemic. Accepting that continued mobility is required for economic sustainability, no country can thrive until all countries can manage their infection rates. As part of that effort, LMICs are now having to choose what subset of mitigating tactics they can afford. Their implementation must be weighed carefully against their feasibility and societal cost. COVID-19 now seems likely to be with us for the long term – that means that effective treatments or vaccines will be critical to solving the crisis. There should be equitable delivery without prejudice, across the globe.

## Author Contributions

NG and HMA conceived and wrote the manuscript. All authors contributed to the article and approved the submitted version.

## Conflict of Interest

The authors declare that the research was conducted in the absence of any commercial or financial relationships that could be construed as a potential conflict of interest.
